# Comparative analysis of widely used methods to remove nonfunctional myosin heads for the in vitro motility assay

**DOI:** 10.1007/s10974-019-09505-1

**Published:** 2019-03-08

**Authors:** Mohammad A. Rahman, Aseem Salhotra, Alf Månsson

**Affiliations:** Department of Chemistry and Biomedical Sciences, Linnaeus University, Kalmar, 391 82 Sweden

**Keywords:** Molecular motor, Myosin, Cross-bridge cycle, In vitro motility assay, Affinity purification, Blocking actin

## Abstract

The in vitro motility assay allows studies of muscle contraction through observation of actin filament propulsion by surface-adsorbed myosin motors or motor fragments isolated from muscle. A possible problem is that motility may be compromised by nonfunctional, “dead”, motors, obtained in the isolation process. Here we investigate the effects on motile function of two approaches designed to eliminate the effects of these dead motors. We first tested the removal of heavy meromyosin (HMM) molecules with ATP-insensitive “dead” heads by pelleting them with actin filaments, using ultracentrifugation in the presence of 1 mM MgATP (“affinity purification”). Alternatively we incubated motility assay flow cells, after HMM surface adsorption, with non-fluorescent “blocking actin” (1 µM) to block the dead heads. Both affinity purification and use of blocking actin increased the fraction of motile filaments compared to control conditions. However, affinity purification significantly reduced the actin sliding speed in five out of seven experiments on silanized surfaces and in one out of four experiments on nitrocellulose surfaces. Similar effects on velocity were not observed with the use of blocking actin. However, a reduced speed was also seen (without affinity purification) if HMM or myosin subfragment 1 was mixed with 1 mM MgATP before and during surface adsorption. We conclude that affinity purification can produce unexpected effects that may complicate the interpretation of in vitro motility assays and other experiments with surface adsorbed HMM, e.g. single molecule mechanics experiments. The presence of MgATP during incubation with myosin motor fragments is critical for the complicating effects.

**Electronic supplementary material:**

The online version of this article (10.1007/s10974-019-09505-1) contains supplementary material, which is available to authorized users.

## Introduction

Cyclic interactions between the molecular motor myosin II and actin filaments underlie cell movement such as muscle contraction. The mechanism of the ATP-driven actin-myosin interaction, as well as several properties of actin and myosin in themselves, may be studied using isolated proteins in the in vitro motility assay (IVMA) (Kron and Spudich 1986). In such studies, isolated myosin or its proteolytic fragments (heavy meromyosin; HMM or Subfragment 1; S1) are adsorbed either to nitrocellulose-coated (Kron et al. 1991) or silanized surfaces (Harada et al. 1990; Fraser and Marston 1995; Sundberg et al. 2003; Albet-Torres et al. 2007). HMM driven sliding of fluorescent actin filaments is then observed in a fluorescence microscope after addition of an MgATP containing assay solution. In addition to being a straightforward method to study key aspects of muscle contraction in vitro the IVMA is useful for studies of disease conditions with mutated proteins [e.g. (Sommese et al. 2013)] as well as drug effects (Straight et al. 2003; Albet-Torres et al. 2009; Rahman et al. 2018). Moreover, the IVMA has also been exploited for development of nanotechnological applications as pioneered in the 1990s (Suzuki et al. 1997; Nicolau et al. 1999). More recently, quite advanced proof of principle devices for biosensing (Lard et al. 2013; Kumar et al. 2016) and bio computation (Nicolau et al. 2016) have been reported.

**Fig. 1:**
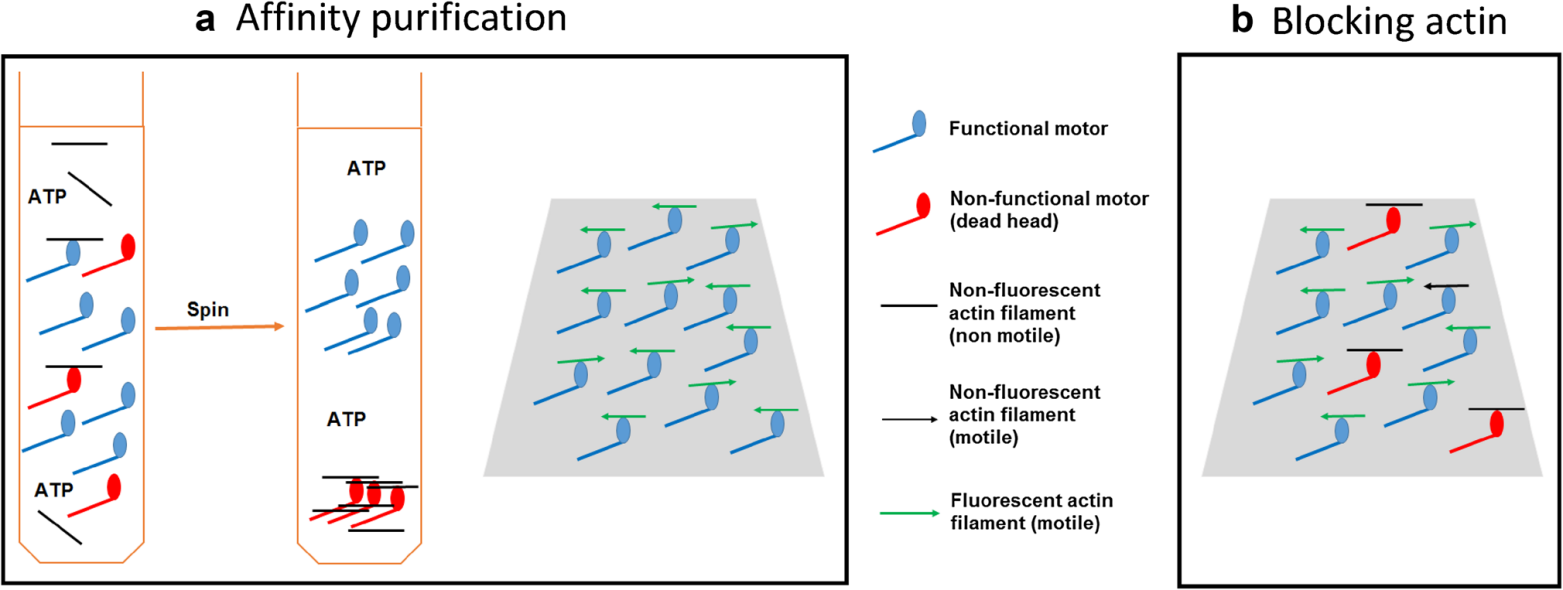
Schematic illustration of the ‘affinity purification’ (left) and ‘blocking actin’ (right) approaches in the IVMA

In a standard IVMA, functional motors propel the actin filaments but a fraction of the heavy meromyosin molecules in a preparation may have nonfunctional heads with ATP insensitive motor domains, e.g. due to oxidation or partial denaturation. These non-functional heads denoted ‘dead heads’ below, act as obstacles against actin sliding. To resolve the problem with dead heads, efforts are often made to remove them or prevent them from interacting with the fluorescent actin filaments. One frequently used approach for removing the dead heads is “actin affinity purification’’ (Kron et al. 1991) simply denoted “affinity purification”, below. In this procedure (Fig. 1a), the myosin motor fragments are mixed with actin filaments and MgATP in solution, followed by ultracentrifugation to pellet any MgATP insensitive motors together with the actin filaments. In an alternative procedure (Fig. 1b), a high concentration of short non-fluorescent actin filaments (here denoted “blocking actin”), are added to surface-adsorbed myosin motor fragments to block the dead heads before adding the fluorescent actin filaments and assay solution. In this approach, the “blocking actin” filaments act as barriers against the interaction between dead heads and fluorescent actin filaments.

Both ‘affinity purification’ and an incubation step with ‘blocking actin’ are procedures commonly used for improving the observed actin-myosin function in the in vitro motility assay. However, the effects of these different approaches on motile properties have not been characterized in any detail. In view of the wide-spread use of the methods, such characterization is important both for appropriate choice between the methods for different purposes and for correct interpretation of experimental results.

In order to achieve this type of characterization, we here performed in vitro motility assays to compare and analyze the effects of ‘affinity purification’ and ‘blocking actin’ on actin-myosin motility in the IVMA. Whereas both procedures increased the fraction of motile filaments to different degrees on different surface substrates we were surprised to find that the actin sliding speed often was reduced by the affinity purification procedure but not by the use of blocking actin. Further experimental tests to analyze the basis for a reduction in velocity suggest that the presence of MgATP during incubation with HMM after “affinity purification” is the key factor, presumably by altering the average HMM conformation and thereby the mode of HMM surface adsorption. This warrants further detailed investigations in the future because different experimental findings may result from different surface adsorption mechanisms of the motors, possibly influencing estimates of myosin step length, stiffness, average force etc.

## Materials and methods

### Materials

All chemicals were of analytical grade and purchased from Sigma Aldrich except Rhodamine Phalloidin that was from Thermo Fisher Scientific (cat. no. R415).

### Ethical statement

Rabbits were sacrificed following an approach that was approved by the regional ethical committee for animal experiments in Linköping, Sweden, reference number 73–14.

### Protein isolation and purification

Heavy meromyosin (HMM) and papain-cleaved myosin subfragment 1 (S1) were prepared and purified from rabbit fast skeletal muscle (Kron et al. 1991). Actin was prepared and purified from rabbit back muscle (Pardee and Spudich 1982). Purity of the proteins was confirmed by sodium dodecyl sulfate polyacrylamide gel electrophoresis (SDS PAGE).

### Buffers and solutions

A low ionic strength solution (Buffer A; pH 7.4) was composed of 10 mM 3-(*N*-morpholino)propanesulfonic acid, 1 mM MgCl_2_ and 0.1 mM Potassium Ethylene glycol-bis(β-aminoethyl ether)-N,N,N´,N´-tetraacetic acid. Assay solution (Buffer B) for the IVMA was prepared in Buffer A with addition of 10 mM DTT, 115 mM KCl, 3 mg/ml Glucose, 0.1 mg/ml glucose oxidase, 0.02 mg/ml catalase, 2.5 mM creatine phosphate, 0.2 mg/ml creatine phosphokinase, 0.64% methylcellulose and 1 mM MgATP (final concentrations). Previous studies (Homsher et al. 1992; Vikhorev et al. 2008) have demonstrated that the addition of methylcellulose to the assay buffer has only minimal effects on velocity. However, it has advantages because it allows the use of ionic strengths close to the physiological values. This prevents fragmentation of actin filaments by strengthening the interaction between the actin subunits (Guan et al. 2005). Wash Buffer (Buffer C) was prepared with 50 mM KCl and 1 mM DTT in Buffer A.

### In vitro motility assays

Standard in vitro motility assays (IVMA) were performed as described earlier (Persson et al. 2013a, b) either on nitrocellulose coated surfaces (Kron et al. 1991) or on surfaces silanized by trimethylchlorosilane (TMCS) (Sundberg et al. 2003; Albet-Torres et al. 2007). Nitrocellulose surfaces were prepared by adding 1% nitrocellulose in amyl acetate to a glass coverslip followed by spreading using a glass rod. The nitrocellulose coated cover glass was then dried in room temperature for 30 min before a flow cell was assembled and used for a motility assay. Surface silanization with TMCS was performed after cleaning the glass coverslips in piranha solution (70% H_2_SO_4_ and 30% H_2_O_2_) at 80 °C for 5 min. Piranha solution was prepared by adding H_2_O_2_ slowly to H_2_SO_4_ (on ice with gentle stirring using a glass rod) and the mixture was heated in a water bath. All the steps, including the preparation of piranha solution and surface silanization, are carried out under the fume hood. (*Caution*: Precaution should be taken while working with Piranha solution. It is a strongly corrosive acidic solution. Explosion may occur in contact with organic materials. Do not store in a closed container). Piranha cleaned glass coverslips were washed in distilled H_2_O (3x; 2 min each) followed by 2 min incubation in methanol, acetone and chloroform, respectively before drying with N_2_ gas flow. Coverslips were then submerged into TMCS (5% v/v in chloroform) solution for 2 min before two final washes (2 min each) in chloroform, followed by drying under N_2_ gas flow. Coverslips, silanized with TMCS, were used within 6 months of preparation.

In the standard IVMA, a flow cell was first incubated with 120 µg/ml HMM (2–5 min) and then with 1 mg/ml BSA (2 min), both in Buffer C. Subsequently, the flow cell was rinsed with Buffer C, incubated with 2–10 nM rhodamine-phalloidin-labeled actin filaments and rinsed again with buffer C prior to addition of the assay solution (Buffer B). Actin filament sliding was recorded and analyzed as described previously (Rahman et al. 2018). An electron multiplying charge coupled device (EMCCD) camera (C9100-12, Hamamatsu Photonics) was used to record the actin filament movements with a frame rate in the range 4–10 frames/s. We used a custom-made MATLAB program (Mansson and Tagerud 2003) to track the actin filament manually by clicking on the leading end of the filament for 15 frames.

To remove MgATP insensitive motor heads by “affinity purification’’, an HMM stock solution was diluted to 120 µg/ml in buffer C. Actin filaments (150 µg/ml; 3.6 µM) were added to HMM and incubated for 5 min followed by MgATP (1 mM) incubation for 5 min. The mixture was then centrifuged at 75,000 RPM (∼ 200 000*g*) using a TLA120.1 rotor (Beckman-Coulter Inc.) in an ultracentrifuge for 10 min at 4 °C [cf.(Kron et al. 1991)] and the supernatant with functional HMM was used immediately in the IVMA. As an alternative approach, 1 µM actin (non-fluorescent “blocking actin” in buffer C) was added to the flow cell after HMM and BSA incubation. After incubation for 2 min, the flow cell was washed one time with buffer C supplied with 1 mM MgATP and then with buffer C alone prior to addition of rhodamine-phalloidin labeled actin filaments and assay solution (buffer B). Analysis of the affinity purification procedure was performed by applying SDS–PAGE to the protein solution before the centrifugation and to the pellet and supernatant after the centrifugation as described in detail in the Supporting Methods, Fig. S1 and Table S1.

In some experiments, 120 µg/ml HMM or S1 was mixed with 1 mM MgATP in buffer C, followed by storage for 40 min at 4 °C, prior to incubation of the IVMA flow cells. For checking the MgATP interaction with surfaces, some flow cells were incubated with 1 mM MgATP in buffer C for 5 min and washed with buffer C (2x) prior to HMM incubation and IVMA using the standard protocol.

**Fig. 2:**
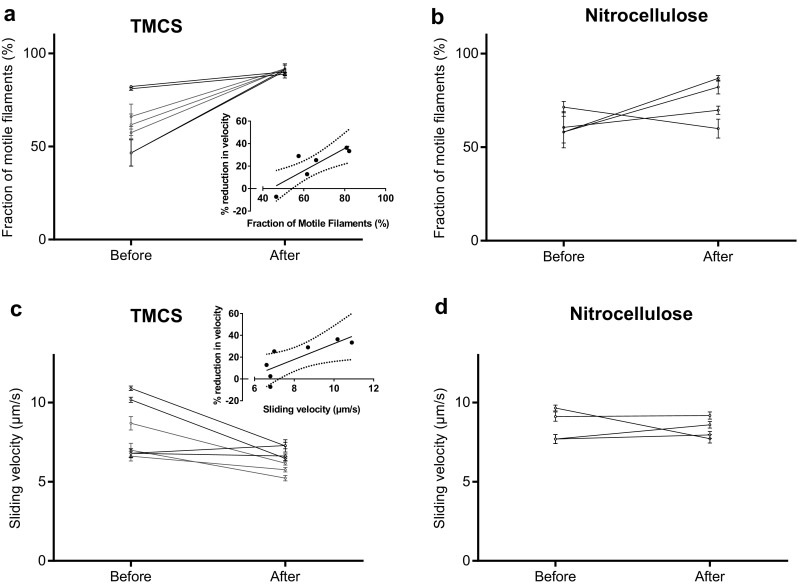
Effect of affinity purification on actin filament motility in the in vitro motility assay. Fraction of motile filaments (%) on **a** trimethylchlorosilane (TMCS) and **b** nitrocellulose surface before and after affinity purification of HMM. Actin sliding speed on **c** TMCS and **d** nitrocellulose surface before and after affinity purification of HMM. The insets in **a** and **c** show the % mean reduction in velocity upon affinity purification as function of the fraction of motile filaments (data in main Fig. 2a) and sliding velocity (data in main Fig. 2c) before the affinity purification. Negative values represent increase in velocity. Regression lines shown with 95% confidence band. Spearman correlation by rank (due to non-Gaussian residual around line) gives statistically significant positive correlation in both **a** and **c** (r = 0.85; p = 0.025). In **c** 12–30 actin filaments were analyzed for each flow cell in the 7 pairs, before and after affinity purification. More details in Fig. S2 and text. In **d** 30 actin filaments were analyzed in pairs as in **c**. The data from 7 different experimental occasions in **a** and **c** and 4 different occasions in **b** and **d**, using 2 different HMM preparations corresponding to 2 different myosin preparations (color coded). Note, more substantial overall difference in behavior (before vs after) between HMM tubes (and flow cells) from a given preparation than between preparations. The temperature was 27.5–28.7 °C on TMCS and 28.5–29.0 °C on nitrocellulose surfaces

**Fig. 3:**
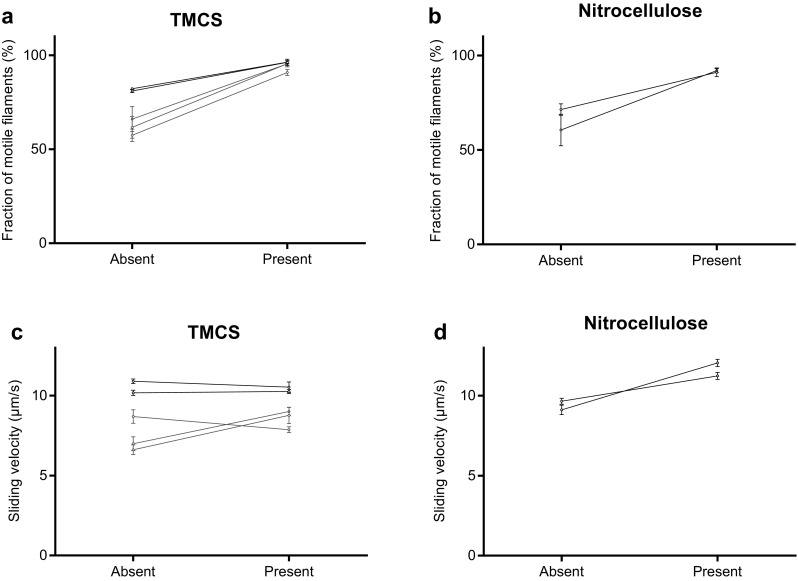
Effect of blocking actin (1 µM) on actin filament motility in the in vitro motility assay. **a** Fraction of motile filaments (%) on TMCS and **b** nitrocellulose surfaces studied in the absence and presence of 1 µM blocking actin. **c** Actin sliding speed on TMCS and **d** nitrocellulose surfaces studied in the absence and presence of 1 µM blocking actin. In **c** 12–30 actin filaments were analyzed in 5 pairs of flow cells either in the absence or presence of blocking actin. In **d** 30 actin filaments were analyzed for each flow cell. The experiments on 5 different occasions were performed using 2 different HMM preparations (each from a different myosin preparation). The myosin preparations are the same as those in Fig. 2 as indicated by color coding. Temperature was 27.5–28.7 °C on TMCS and 28.5–29.0 °C on nitrocellulose surfaces

**Fig. 4:**
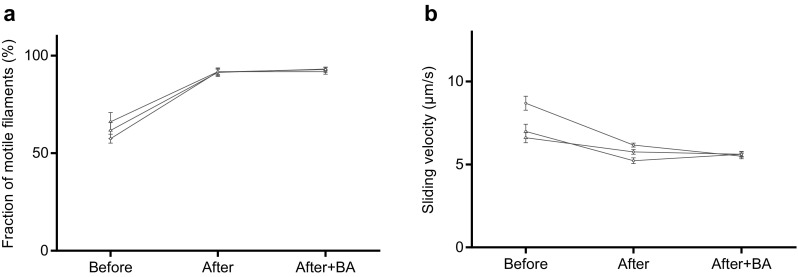
Comparison between effects of blocking actin and affinity purification using both procedures in series on TMCS surfaces. **a** Fraction of motile filaments and **b** sliding velocity before affinity purification, after affinity purification and after affinity purification and addition of blocking actin (BA). In **b** 12**–**30 actin filaments were analyzed for each flow cell using 3 different HMM tubes from 1 HMM preparation. Experiments before and after affinity purification also included in Fig. 2 where it can be seen that the effects of affinity purification are representative. Same HMM tubes also used for analysis of affinity purification procedure by SDS–PAGE (cf. Figs. S1, S2). Temperature, 27.8–28.2 °C

**Fig. 5:**
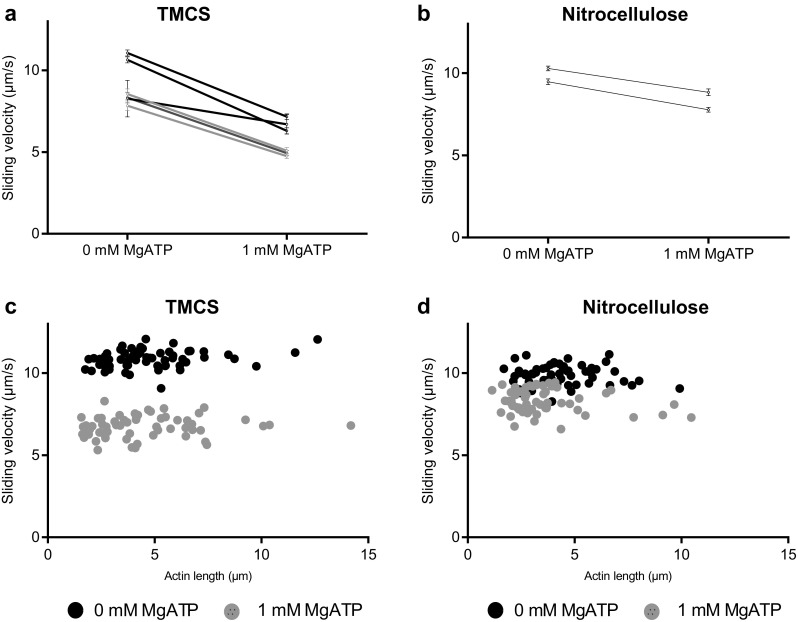
Effect of the presence of MgATP (1 mM) in the HMM (120 µg/ml) incubation solution on actin sliding speed in the in vitro motility assay. **a** Actin sliding speed on TMCS and **b** nitrocellulose surface. **c** Actin sliding speed for individual filaments plotted against actin filament length on TMCS surface and **d** nitrocellulose surface. The data are for experiments in **a** and **b** with large reduction in velocity following HMM incubation in the presence of MgATP. In **a** 7–30 actin filaments were analyzed for each flow cell out of 6 pairs. In **d** 30 actin filaments were analyzed for each flow cell. In these experiments, on different occasions, we used 3 different HMM preparations (each from a different myosin preparation). These preparations are those used above as indicated by color coding and one further preparation (blue). Note very similar overall effects of the presence of MgATP during HMM incubation for different HMM preparations despite differences in sliding velocity. The latter difference is attributed to different fraction of dead heads (cf. Fig. 3a, c), ∼ 1 °C lower temperature in experiments with “blue” and “red” preparations (∼ 28 °C) compared to the “black preparation” (∼ 29 °C) and other factors (< 10%). The latter include possible differences between solutions, surfaces, myosin isoform composition etc. Temperature was 27.8–29.2 °C on TMCS and 28.6–29.0 °C on nitrocellulose surfaces. (Color figure online)

**Fig. 6:**
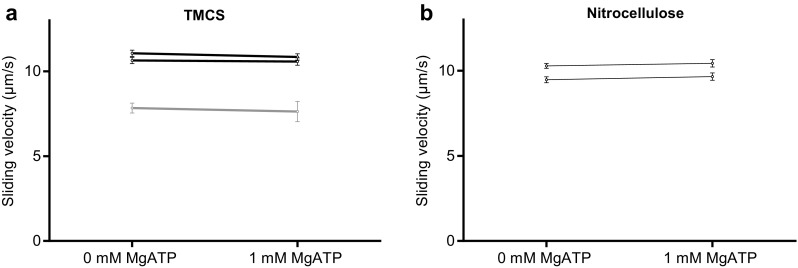
Effect of MgATP (1 mM) incubation of surface prior to incubation with HMM for an in vitro motility assay. **a** Actin sliding speed on TMCS and **b** nitrocellulose surfaces. In **a** 10–30 actin filaments were analyzed for each flow cell in a pair. Two different HMM preparations (2 Myosin preparations) were used. Color coding as in Fig. 5. In **b** 30 actin filaments were analyzed for each flow cell using 2 different HMM tubes from 1 HMM preparation. Temperature was 27.8–29.2 °C on TMCS and 28.6–29.0 °C on nitrocellulose surfaces. (Color figure online)

**Fig. 7:**
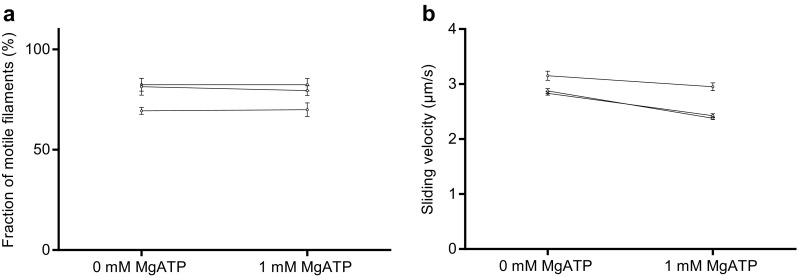
Effect of the presence of MgATP (1 mM) in the S1 (120 µg/ml) incubation solution on fraction of motile filaments and actin sliding speed in the in vitro motility assay on TMCS surface. **a** Fraction of motile filaments and **b** Sliding velocity. In **b** 15–30 actin filaments were analyzed for each flow cell using 2 different S1 preparations (2 different myosin preparations). Temperature was 27.9–28.4 °C

Altogether we used HMM preparations from three different myosin preparations in the in vitro motility assay experiments (Figs. 2, 3, 4, 5, 6, 7). Notably, the difference in observed behaviour varied less between the different HMM preparations than between different experiments using a given HMM preparation (cf. Figs. 2, 5 below).

Actin sliding velocity was characterized by the coefficient of variation (CV) (standard deviation of the frame-to-frame velocity divided by average velocity) for 10 frames during the analysis. The velocity data that were used in the study was limited to filament paths with CV < 0.2 (Mansson and Tagerud 2003). Linear regression was performed using Graph Pad Prism (v. 7.03; GraphPad Software, San Diego, CA) that was also used for other statistical analyses. Data are given as mean ± 95% Confidence Interval (95% CI) unless otherwise specified.

## Results

First, we performed in vitro motility assays using ‘affinity purification’ to remove MgATP insensitive “dead” motors prior to HMM incubation of the motility assay flow cells. In accordance with expectations (Homsher et al. 1992; Fraser and Marston 1995; Winkelmann et al. 1995), affinity purification of the HMM preparation significantly increased the fraction of motile filaments. The effect was observed in all experiments using TMCS surfaces (Fig. 2a) where the fraction of motile filaments increased to ∼ 90% (89–92%) independent of the fraction of motile filaments (47–82%) observed without affinity purification. The effect was also seen in 3 out of 4 experiments on nitrocellulose but with overall greater variability between experiments (Fig. 2b). On TMCS, the increase in the fraction of motile filaments was seen even if the fraction was high (82%) in the absence of affinity purification. However, the largest effect was observed for the cases where lowest fraction of motile filaments was observed in the absence of affinity purification. A surprising result was the finding that affinity purification significantly reduced the actin sliding speed in five out of seven experiments on TMCS (Fig. 2c) and one out of four experiments on nitrocellulose surfaces (Fig. 2d) as indicated by the 95% CIs in Fig. 2. This effect was analyzed in greater detail for TMCS surfaces showing that the reduction in velocity is positively correlated with both the fraction of motile filaments and the sliding velocity before the affinity purification (insets of Fig. 2a, c). It is also indicated by the color coding in Fig. 2 that the effect of affinity purification was similar overall for experiments using either of two different HMM (and myosin) preparations. See further Materials and Methods and Fig. 5 with regard to similarity in behavior between different myosin preparations.

We also tested the addition of blocking actin after HMM adsorption as an alternative to affinity purification for increasing the fraction of motile filaments. This approach significantly increased the fraction of motile filaments both on TMCS (Fig. 3a) and nitrocellulose surfaces (Fig. 3b), also if this fraction was high in the absence of blocking actin. In contrast to the effect of affinity purification, the actin sliding speed was negligibly changed by use of blocking actin in three experiments on TMCS surfaces (Fig. 3c) and significantly increased in two experiments each on TMCS and nitrocellulose surfaces (Fig. 3d). The difference in effects of blocking actin and affinity purification on velocity is further substantiated by direct comparison (Figs. S2–S3) of results from three different experiments with one HMM preparation (subsets of data for TMCS in Figs. 2, 3). In these experiments the effect of blocking actin and affinity purification was studied in parallel for three HMM tubes on three different experimental occasions using a given assay solution on each occasion.

Using the same HMM tube as in Figs. S2–S3, we also found (Fig. 4a) that subsequent addition of blocking actin, following affinity purification, did not change the fraction of motile filaments compared to that seen after affinity purification alone. Neither was the sliding velocity further changed by this procedure (Fig. 4b).

The above results are consistent with two mechanisms mediating the effects of affinity purification, one mechanism that tends to reduce velocity without an effect on the fraction of motile filaments and one mechanism that tends to increase both the fraction of motile filaments and velocity. The latter mechanism is readily identified with the removal of dead heads because it dominates when the fraction of motile filaments and the velocity are initially low indicating a large fraction of dead heads (cf. Fig. 2). We attribute the second mechanism, tending to reduce velocity, to other effects of the affinity purification than removal of dead heads. Possibilities include removal of functional myosin heads or altered surface adsorption mechanisms, and thereby altered actin propelling capability, for the HMM molecules subjected to the affinity purification procedure.

The fact that addition of blocking actin after affinity purification (Fig. 4) neither modified the effect of affinity purification on velocity nor on the fraction of motile filaments is consistent with the following ideas: (1) Affinity purification effectively removes the “dead” myosin heads because no further improvement is achieved with addition of blocking actin and (2) The reduction in velocity upon affinity purification is not due to a reduced surface density of actin-binding myosin motors. If that had been the case, addition of blocking actin (1 µM) would be expected to further reduce velocity due to competition for myosin heads (cf. Fig. S4) with the fluorescent actin filaments (< 10 nM) that are the ones actually analyzed in the assay. The evidence for negligible removal of functional myosin heads by the affinity purification procedure is supported by the observation (Supporting Information) that less than ∼ 12% (range 5–12%) of the myosin heads were lost into the pellet during the affinity purification procedure in three different experiments and that a substantial fraction of those heads were non-functional as indicated by significantly increased fraction of motile filaments from 57.5 to 66% without affinity purification to ∼ 90% after the procedure (Fig. S2).

We and others (Albet-Torres et al. 2007; Nicolau et al. 2007; Lindberg et al. 2018) have previously found that varied surface hydrophobicities, reflected in varied surface contact angle in the range 0–85°, alter the actin sliding velocity, in a graded fashion, from zero at the lowest contact angles to a maximum at contact angles > 70° (as on the TMCS derivatized surfaces used here). This effect has been attributed to gradual variation in the distribution between different surface adsorption configurations of HMM with varying contact angle (Albet-Torres et al. 2007; Persson et al. 2010; Månsson 2012; van Zalinge et al. 2012; Lindberg et al. 2018). It therefore seems likely that small differences in the HMM conformation e.g. with and without nucleotide at the active site, also may alter the HMM adsorption mechanism. We thus considered the possibility that the reduction in sliding speed upon affinity purification is due to effects of MgATP in the HMM incubation solution that modify the surface adsorption mechanism. This idea was tested by mixing HMM with 1 mM MgATP followed by storing (without ultracentrifugation) for 40 min at 4 °C prior to incubation of motility assay surface. Interestingly, subsequent motility assays showed that the actin sliding speed was consistently reduced in all experiments on both TMCS (Fig. 5a) and nitrocellulose surfaces (Fig. 5b) compared to motility assays without MgATP in the HMM incubation solution. Because both affinity purification and MgATP incubation with HMM resulted in reduction of actin sliding speed, it seems that the presence of MgATP during HMM incubation is central for the effect. This idea is also consistent with the findings that the addition of blocking actin did not reduce velocity (Figs. 3, 4).

One possible basis for lower speed in the presence of 1 mM MgATP could be a lower HMM motor density on the surface due to reduced surface adsorption of HMM in actin-binding conformations in the presence of MgATP. However, if the motor density is reduced to a degree that the speed is appreciably affected one would expect an increased sensitivity of speed to changes in the number of myosin heads that are available for interaction with the actin filaments (Toyoshima et al. 1990; Uyeda et al. 1990). As a special case, one would expect increased speed reduction with reduced filament length in the in vitro motility assay (Uyeda et al. 1990; Persson et al. 2013a, b). Importantly, however, such an effect was not observed. Instead, the relationships between actin sliding speed and actin filament length (Fig. 5c, d; Fig. S5) show that both the shortest and longest filaments moved at similar mean speeds. These results are consistent with the idea that the reduced actin sliding speed, observed when HMM is incubated with 1 mM MgATP, is not due to lower density of actin-binding myosin heads on the surface. The alternative possibility seems to be that the HMM molecules in the presence of MgATP adsorb to the surface at similar density and in a configuration with similar actin binding capacity as in the absence of MgATP but with lower capability to produce sliding at high velocity. We hypothesized that this is either due to pre-binding of MgATP to the surface before the HMM adsorption or to another configuration of the HMM molecule when nucleotide and inorganic phosphate (Pi) are bound to the active site in the presence of MgATP. To distinguish between these possibilities, we first incubated the motility assay surfaces with 1 mM MgATP (5 min) prior to incubation with HMM (without affinity purification and without MgATP). In this case, the sliding speed was similar in all experiments both on TMCS (Fig. 6a) and on nitrocellulose surfaces (Fig. 6b) as in the complete absence of MgATP, arguing against the relevance of MgATP interaction with the surface prior to HMM adsorption. This lead us to conclude that the reduction in velocity when MgATP is present during HMM incubation is attributed to the binding of substrate and/or product (MgADP and Pi) to the active site of myosin.

In order to further elucidate the molecular mechanisms behind the reduction in velocity when the motility assay surfaces are incubated with HMM in the presence of MgATP we also studied the effect on motility assays using myosin subfragment 1 (S1) when incubated either in the presence or absence of MgATP. Whereas the fraction of motile filaments was similar whether MgATP was present or not (similar as with HMM; not shown), the effect on the velocity was qualitatively similar to that seen with HMM (Fig. 7). That is, the velocity was significantly reduced (as indicated by non-overlapping 95% confidence intervals) in all three experiments although the effect of MgATP was quantitatively smaller than the reduction in velocity generally seen with HMM (Fig. 5a). The mechanistic basis for the results is discussed below.

## Discussion

The in vitro motility assay is widely used for studies of molecular motor function and for nanotechnological applications (see “Introduction’’). In order to improve motile function in the assay, ATP insensitive motors are often removed by an affinity purification method similar to that used here (Kron et al. 1991; Homsher et al. 1992; Fraser and Marston 1995; Winkelmann et al. 1995; Persson et al. 2013a, b; Sommese et al. 2013). This procedure leads to incubation conditions where the myosin motor fragments are added to the motility assay surface in the presence of millimolar [MgATP]. This is different from conventional motility assays without affinity purification where MgATP is not generally present during motor adsorption. To the best of our knowledge, it has not been studied previously how the presence of MgATP during HMM incubation might affect function. Here we investigated this issue while comparing the affinity purification procedure to the use of blocking actin with regard to improvement in motile function in the in vitro motility assays with HMM adsorbed to TMCS-derivatized or nitrocellulose coated glass surfaces. Whereas both procedures increased the fraction of motile filaments, our results show that the affinity purification procedure also has the undesirable potential to reduce sliding velocity. A similar effect was not seen with the use of blocking actin. Based on our results it seems that reduced velocity after affinity purification is primarily seen on TMCS derivatized surfaces. However, our evidence (Fig. 5b) suggests that the presence of MgATP during HMM incubation also tends to reduce velocity on nitrocellulose surfaces. Overall, the findings seem to reflect the existence of two opposing effects: (1) an increase in velocity due to removal of dead heads or blocking of them and (2) a reduction in velocity associated with incubation of HMM in the presence of MgATP. The idea of two opposing effects is consistent with a positive correlation between the magnitude of the velocity reduction on TMCS and the fraction of motile filaments before affinity purification, including the increase in velocity in one case when the fraction of motile filaments was initially low on TMCS. In the latter case, the increase in velocity due to removal of dead heads dominates over the second velocity reducing mechanism. The idea of two opposing effects is also consistent with the effects on sliding velocity seen on nitrocellulose surfaces, with different effects of affinity purification (Fig. 2d), blocking actin (Fig. 3d) and HMM incubation in the presence of MgATP (Fig. 5b). In the first case (Fig. 2d), the two factors (1 and 2 above) outbalance each other with minimal changes in velocity. In the second case (Fig. 3d) only the velocity-increasing effect (eliminated influence of dead heads) is present. Finally, in the third case (Fig. 5b), only the second velocity reducing effect is present.

Our studies accord with the view that HMM, in the presence of MgATP (“HMM-ATP” below), adsorbs to motility assay surfaces in a configuration that produces reduced actin sliding speeds compared to the situation without MgATP. However, the velocity–length plots (Fig. 5c, d) and the lack of effects of blocking actin after affinity purification (Fig. 4) provide evidence against reduced surface-adsorption of HMM–ATP and/or against reduced capability of the HMM molecules to bind to actin filaments. Nevertheless, the presence of MgATP during HMM incubation seems to be the critical factor behind the reduction in velocity. This is suggested by results of the experiments where MgATP was mixed with HMM without affinity purification. During such incubation of HMM in the presence of excess MgATP, HMM has primarily MgADP and Pi (inorganic phosphate) at the active sites. The possibility that accumulation of MgADP in the assay solution during pre-incubation and surface incubation should underlie a reduced sliding speed (Homsher et al. 1992) is highly unlikely because there are several rinsing steps between the HMM incubation and the actual motility assay. These rinsing steps would effectively remove any ATP hydrolysis products added together with HMM, arguing strongly against speed reduction due to the presence of MgADP. Instead, the simplest interpretation of our results is that the Pi and MgADP occupation of the myosin active site during HMM incubation changes the distribution between different HMM configurations on the surface. The lack of effects on velocity of blocking actin after affinity purification (Fig. 4b) and the unaltered velocity–length plots with HMM–ATP (Fig. 5c–d; Fig. S5) suggest that the change in distribution between different HMM configurations only negligibly reduces the fraction of actin-interacting myosin heads.

**Fig. 8:**
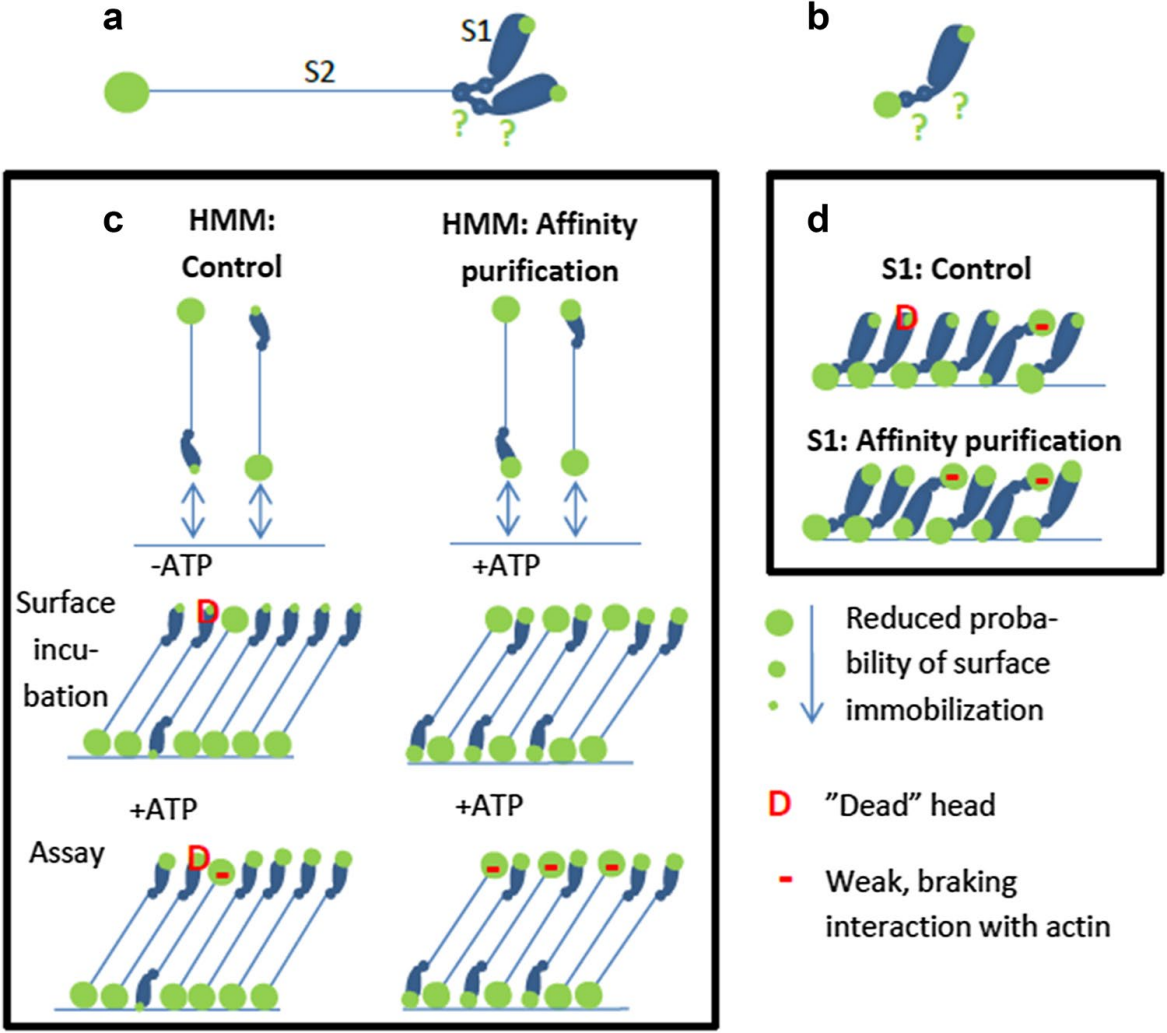
Model for adsorption of heavy meromyosin (HMM) and subfragment 1 (S1) to surfaces. **a** Schematic illustration of HMM with two motor domains (S1; heads) to the right and the coiled–coiled subfragment 2 (S2) domain to the left. Green circles indicate most likely sites for adsorption to a TMCS surface. The experimental and theoretical basis for the model with high probability of adsorption via the C-terminal tail (chymotrypsin cleavage site; large circle) and lower probability via the actin-binding region (small circle) is given in detail previously (Albet-Torres et al. 2007; Månsson et al. 2008; Persson et al. 2010; Månsson 2012; Lindberg et al. 2018). Question marks indicate the possibility of further interaction sites between surfaces and HMM. **b** Schematic illustration of S1 domain assuming greatest probability of adsorption via the papain cleavage site at the C-terminal end of S1. **c** At high HMM incubation concentration, the HMM molecules are most likely to approach the surface either via the C-terminal tail domain or the head domain (Månsson et al. 2008). If the surface approach leads to adsorption or not depends on the binding probability at the approaching end of the molecule. Because the probability is greatest for the C-terminal tail, a larger fraction of the molecules will adsorb via the tail as indicated in lower two rows of the panel. According to our model, affinity purification (right part of panel) not only removes HMM with dead heads (red; “D”) but the binding of MgADP and Pi to the active site of myosin leads to increased probability of adsorption via the head domain, increasing the fraction of the HMM molecules that are adsorbed via the heads. **d** Model for the effect of affinity purification on S1 adsorption to surface. In the present work we only studied the MgATP effect that we assume increases the probability of S1-adsorption to the surface via the actin-binding site

We consider our results in relation to a model based on experimental data for HMM adsorption with close to saturating HMM densities on TMCS derivatized surfaces (Sundberg et al. 2006; Albet-Torres et al. 2007; Månsson et al. 2008; Persson et al. 2010; Månsson 2012; Lindberg et al. 2018). According to this model, HMM at high solution concentration (> 100 µg/ml) adsorbs to the surface either via the actin-binding region or the C-terminal chymotrypsin cleavage site of S2 (Fig. 8) and holds the actin filament more than 30 nm above the surface (Persson et al. 2010). These characteristics suggest that any changes in surface adsorption location between different sites on the catalytic domains [heads; (Toyoshima 1993)] of HMM would not affect motility because the heads do not reach the actin filament > 30 nm above the surface. The only reasonable mechanism is instead a small increase in the fraction of the HMM molecules that adsorb via the head region. These HMM molecules must encompass a small enough fraction of all myosin heads to maintain the number of actin-interacting heads at a saturating level for actin filament velocity. Instead we attribute the velocity reduction to braking interactions between the C-terminal tail region and actin. This idea is consistent with a reduction in actin sliding velocity on surfaces of reduced contact angle (Albet-Torres et al. 2007; Persson et al. 2010; Månsson 2012; Lindberg et al. 2018) where the effect has been attributed to an increased fraction of HMM molecules with the tail regions extending away from the surface towards the actin filaments. The effect may also be related to the finding (Guo and Guilford 2004) that the presence of the coiled-coil light meromyosin fragment of myosin in an in vitro motility assay reduces sliding velocity. This fragment contains the other end of the chymotryptic cleavage site that would be exposed by head-adsorbed HMM molecules.

The idea put forward above requires structural changes in the myosin head region upon binding of MgADP and Pi that increases the probability of adsorption via this site because adsorption via the C-terminal site is unlikely to be affected. This idea (almost self-evident in view of the energy transduction by the myosin motor domain) is consistent with well-known structural changes in the motor domain upon nucleotide binding and hydrolysis (Highsmith and Eden 1990; Sugimoto et al. 1995; Duxbury et al. 2004; Thomas et al. 2009; Sato et al. 2016), e.g. changed electrostatic surface potential (Sato et al. 2016) and altered shape (Sugimoto et al. 1995; Thomas et al. 2009). The former change may modulate electrostatically driven adsorption to TMCS-derivatized surfaces (Albet-Torres et al. 2007). The changes in shape, on the other hand, could be the basis for exposure of additional hydrophobic sites, possibly triggering adsorption associated with partial unfolding of the motor domain (Månsson et al. 2008). The mentioned mechanisms are also consistent with reduced sliding velocity when S1 adsorbs to TMCS in the presence of MgATP suggesting a limited switch among the adsorbing configurations from immobilization via the C-terminal end to the actin-binding region of S1. Experiments using different affinity tags to attach HMM to the surface (e.g. antibodies to certain regions of the molecule) (Winkelmann et al. 1995; Sommese et al. 2013) may be useful to further elucidate how the presence of MgATP during HMM incubation affects velocity. However, such studies are outside the scope of the present work because of additional complexities. For instance, the studies would require full characterization of possible non-specific actin-interactions that may differ between antibodies as well as the selectivity of the antibodies against different epitopes on HMM and possible changes of the selectivity in the presence of MgATP.

It is of interest to consider how the differences between TMCS and nitrocellulose fit into the picture depicted above. The major differences are: (1) reduced tendency for velocity reduction on nitrocellulose than on TMCS following the affinity purification process (Fig. 2c–d) and (2) increased sliding velocity on nitrocellulose but not on TMCS with use of blocking actin if the velocity was high (> 9 µm/s) without blocking actin. It seems likely that these differences are related to different surface adsorption mechanisms of HMM on TMCS and nitrocellulose (Albet-Torres et al. 2007; Hanson et al. 2017). Whereas HMM primarily seems to be adsorbed via the C-terminal tail domain to TMCS (Albet-Torres et al. 2007; Balaz et al. 2007; Persson et al. 2010), there is evidence (Toyoshima 1993; Winkelmann et al. 1995) that HMM molecules are tethered to nitrocellulose in several configurations. A different distribution between adsorption via different sites on HMM to TMCS and nitrocellulose seems reasonable in view of chemical and physical differences between the two surface substrates. Whereas the surfaces exhibit similar contact angles, TMCS derivatized glass is a hard surface substrate dominated by methyl groups and charged SiO groups (Albet-Torres et al. 2007), and nitrocellulose is a “soft” surface with certain roughness (Sundberg et al. 2003; Hanson et al. 2017). Nitrocellulose also has a wider range of different functional groups which may interact with different parts of HMM (cf. discussion in (Albet-Torres et al. 2007) and references therein).

It is of interest to consider the present results in relation to variability between different single molecule studies in key myosin properties such as step length, actomyosin stiffness and cross-bridge force (Kaya and Higuchi 2013; Månsson et al. 2018). These differences are largely attributed to the use of different types of single molecule preparations and different details of the force-measurement techniques and analyses (Kaya and Higuchi 2013; Månsson et al. 2018). However, the possibility should also be considered that part of the variation arises because affinity purification has been used in some studies but not in others. Similar effects also need to be considered when comparing maximum sliding velocities in the in vitro motility assay from different studies under seemingly similar conditions. Finally, it is relevant to mention that effects of the affinity purification on functional properties of surface immobilized motors may possibly only occur if myosin is non-specifically adsorbed to the surface. If the motor fragment is bound to the surface via specific affinity tags such as antibodies against the S2 domain (Winkelmann et al. 1995) or against a co-expressed green fluorescent protein (GFP) moiety (Sommese et al. 2013), effects on the motor structure of MgADP and Pi at the active site may be less likely to affect the surface binding mode. On the other hand, one may foresee that such secondary surface immobilization may give lower density of motors. This could possibly reduce velocity in the in vitro motility assay upon addition of blocking actin due to competition with fluorescent actin for the available myosin motors. Furthermore, it cannot be fully excluded that the affinity of antibodies to different epitopes on myosin motor fragments may vary depending on whether MgATP is present or not. A consideration when using blocking actin at standard concentration of 1 µM is the tendency of motile actin filaments at high densities (Fig. S4b) to exhibit swarm-like behavior [cf. (Kraikivski et al. 2006; Vikhorev et al. 2008a, b; Butt et al. 2010; Schaller et al. 2010)]. This could confound the results of some in vitro motility assay studies where random updates in actin filament sliding direction are essential such as in efforts to characterize the actin filament flexural rigidity (Bengtsson et al. 2016). Importantly, however, the potential of swarm-like motion did not seem to affect velocity and fraction of motile filaments recorded in the presence of blocking actin in the present study (cf. Fig. 4).

## Electronic supplementary material

Below is the link to the electronic supplementary material.

Supplementary material 1 (DOCX 926 KB)

Supplementary material 2 (AVI 3648 KB)

Supplementary material 3 (AVI 4147 KB)

Supplementary material 4 (AVI 3210 KB)

Supplementary material 5 (AVI 3383 KB)

Supplementary material 6 (AVI 3670 KB)

Supplementary material 7 (AVI 4458 KB)

Supplementary material 8 (AVI 3722 KB)

Supplementary material 9 (AVI 3778 KB)

Supplementary material 10 (AVI 1376 KB)
