# Supplementary material for: Comparative analysis of widely used methods to remove nonfunctional myosin heads for the in vitro motility assay
Source: J Muscle Res Cell Motil. 2019 Mar 8;39(5):175–87. doi: 10.1007/s10974-019-09505-1 (PMC6494787; doi:10.1007/s10974-019-09505-1)
Supplement: Supplementary file 1 — Supplementary material 1 (DOCX 926 KB) [file 10974_2019_9505_MOESM1_ESM.docx]

**Comparative analysis of widely used methods to remove nonfunctional myosin heads for the in vitro motility assay**

by

# Mohammad A. Rahman, Aseem Salhotra and Alf Månsson

Department of Chemistry and Biomedical Sciences, Linnaeus University, SE-391 82 Kalmar, Sweden.

Supporting Method

**Estimation of fraction of HMM lost in affinity purification process**

We performed affinity purification using 3 randomly selected HMM tubes following the protocol of (Kron, Toyoshima, Uyeda, & Spudich, 1991). After the affinity purification we recovered the supernatant containing functional HMMs in a fresh tube and washed the pellet with wash buffer (50 mM KCl and 1 mM DTT in a low ionic strength solution (pH 7.4) that was composed of 10 mM 3-(N-morpholino)propanesulfonic acid, 1 mM MgCl_2_ and 0.1 mM Potassium Ethylene glycol-bis(β-aminoethyl ether)-N,N,N´,N´-tetra acetic acid). The pellet was then air dried on ice and dissolved directly in sodium dodecyl sulfate polyacrylamide gel electrophoresis (SDS PAGE) sample buffer for SDS PAGE using the standard protocol from the manufacturer (Thermo Fisher Scientific Inc.). In the gel, we loaded a known concentration of HMM as a control, the supernatant containing functional HMM after affinity purification, the full pellet and the pellet wash buffer for SDS-PAGE. After performing the electrophoresis, the gel was stained with a commercial gel staining kit (NOVEX Colloidal Blue Staining Kit from Thermo Fisher Scientific Inc) and imaged using a computer scanner. Protein concentrations were calculated from the protein band intensity analysis using Image J (Image J 1.52i; National Institute of Health, USA). The amount of pelleted HMM in the affinity purification is calculated in relation to the total amount protein from the supernatant, pellet and pellet wash buffer. The gel analysis shows that ~12 % or lower fraction of the HMM was pelleted. The results are summarized in the Table S1. The affinity purification procedures described in Fig. S1 and Table S1 gave representative results (Figs. S2-S3) in the in vitro motility assay experiments (cf. Main Fig. 2, 4).

Supporting Figures

5. Actin

4. Full pellet

3. Pellet wash buffer

2. Supernatant

1. HMM, standard


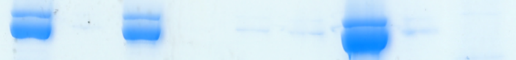

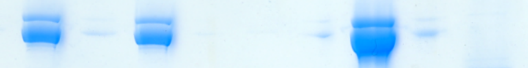


Gel 2

Gel 1

**Fig. S1. Gel (4-20% Tris-Glycine Gel) images after SDS PAGE.** Image limited to the area of the gel corresponding to the molecular mass of HMM. Samples are loaded into the gel as follows: 1. HMM (standard; 1.5µg before affinity purification), 2. supernatant, 14µl out of 500 µl total volume after affinity purification, 3. pellet wash buffer, 14µl from 500 µl wash buffer used for washing the pellet to remove any unwanted functional HMM in unstirred layer close to the centrifuge tube wall), full pellet (air dried on ice after washing with buffer and then dissolved directly in sample buffer) and actin (58.8 µg of actin stock). The gels were labeled with Colloidal Blue Staining Kit from Thermo Fisher Scientific Inc and scanned with a computer scanner.


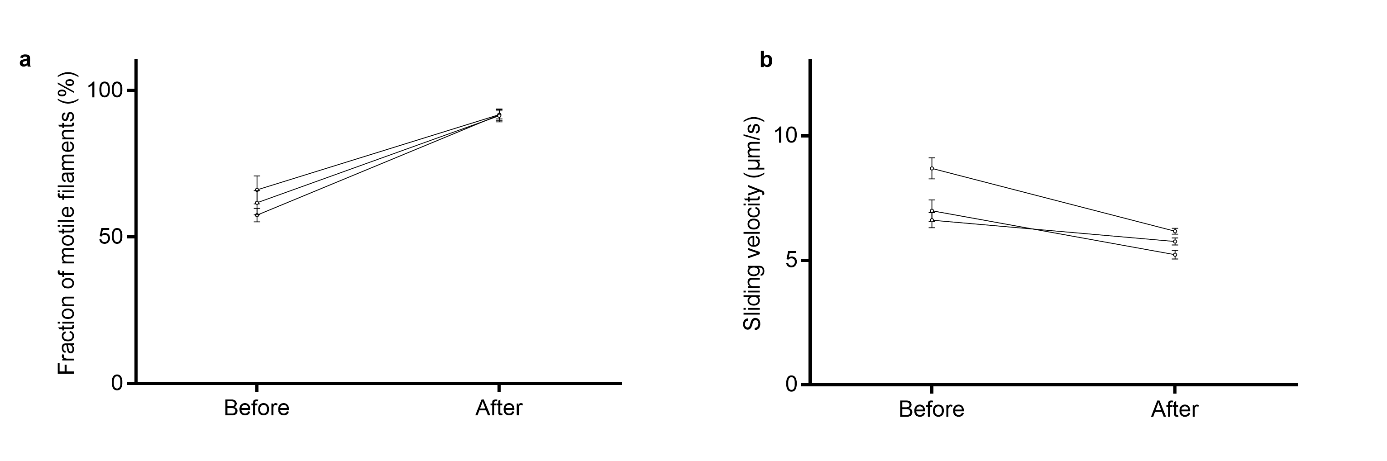


**Fig. S2.** **Effect of affinity purification on actin filament motility in the in vitro motility assay on TMCS surfaces. a** Fraction of motile filaments (%) and **b** Actin sliding velocity studied before and after the affinity purification procedure. In **b** 12-30 actin filaments were analyzed for each flow cell. The three experiments were performed on three occasions using one myosin preparation derived from the three tubes used for SDS PAGE analysis in Fig. S1 and Table S1. Data are also shown in the main Figs. 2 and 4. Temperature was 28.1-28.7^0^C.


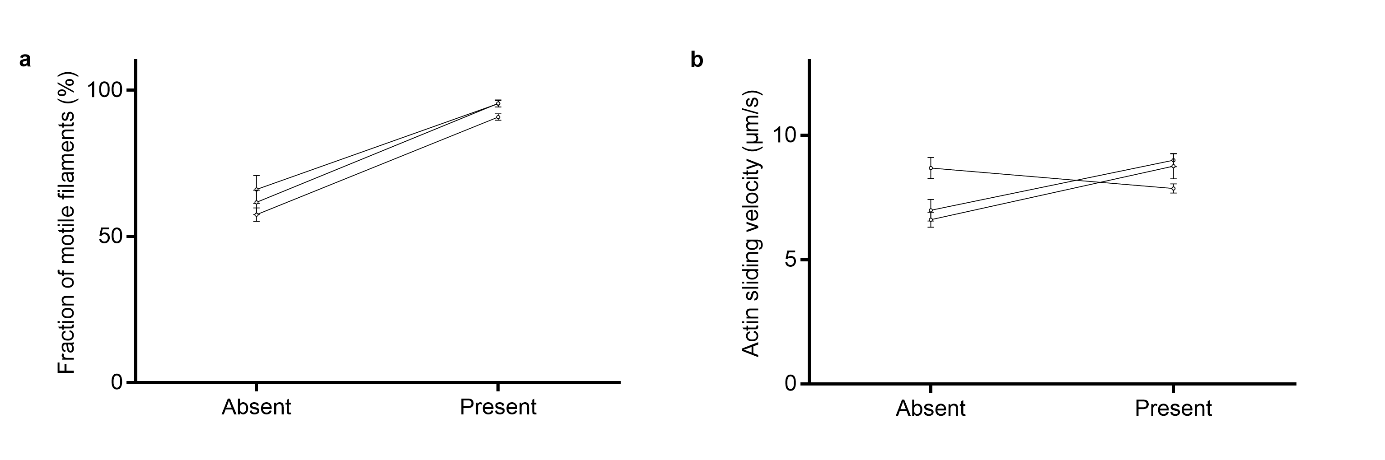


**Fig. S3.** **Effect of blocking actin (1µM) on actin filament motility in the in vitro motility assay on TMCS surfaces. a** Fraction of motile filaments (%) and **b** Actin sliding velocity (µm/s) studied in the absence and presence of 1µM blocking actin. In **b** 12-18 actin filaments were analyzed for each flow cell. Three HMM from one myosin preparation were used on three different experimental occasions. The three experiments were performed on three occasions in parallel with those in Fig. S2, using one myosin preparation derived from the three tubes used for SDS PAGE analysis in Fig. S1 and Table S1. Data are also included in the main Fig. 3. Temperature was 28.1-28.7^0^C.


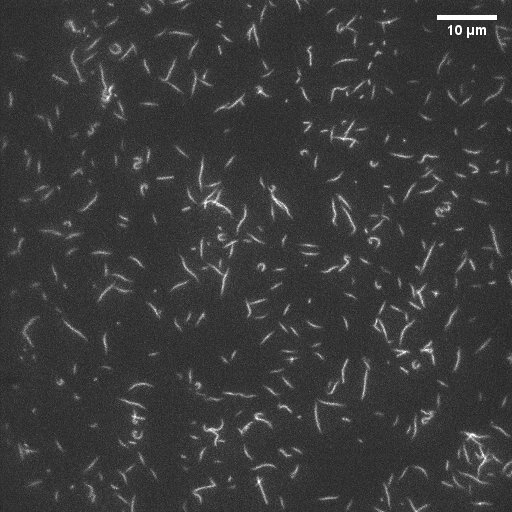

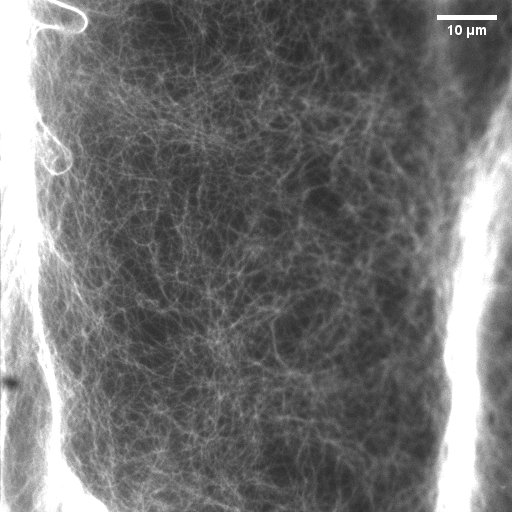


**b**

**a**

**Fig. S4. Fluorescence micrograph showing blocking actin labelled with Alexa-488 phalloidin in one control experiment. a** 20 nM and **b** 1µM Blocking actin (see also Movie S9). The sliding of Rhodamine-phalloidin labelled actin filaments 10 nM analyzed as in other experiments where blocking actin is not fluorescence labelled**.** Scale bar = 10 µm. Experiment on TMCS derivatized surface.

**Fig S5 Effects of the presence of MgATP (1mM) in the HMM (120µg/ml) incubation solution on actin sliding speed in the in vitro motility assay.** Actin sliding speed plotted against actin filament length for all analyzed filaments in all experiments in main Fig. 5a,b. **a-f** TMCS surfaces and **g-h** nitrocellulose surfaces. More details in the legend of Fig. 5. It may be noted that temperature was ~29 ^o^C in **d**, **e**, **f,** and **g** but ~28 ^o^C in the remaining experiments. Further, note that drop in velocity for short filaments occur, if anything, at longer lengths when MgATP is absent during HMM incubation than when it is present. This is consistent with the idea that, if anything, the density of actin-binding myosin motors is higher when MgATP was present.

Supporting Table

**Table S1. HMM quantification from SDS PAGE (cf. Fig. S1) before and after affinity purification. Image of Gel 3 is not shown in Fig. S1.**

|  | Gel 1 | Gel 2 | Gel 3 |
| --- | --- | --- | --- |
| HMM in pellet (HMM_pellet) | 6.61 µg | 2.72 µg | 2.95 µg |
| HMM in pellet wash buffer | 0.80 µg | 0.21µg | N/A |
| HMM in Supernatant (HMM_sup) | 47.19 µg | 51.96 µg | 48.30 µg |
| Total HMM | 54.61 µg | 54.90 µg | 51.24 µg |
| % HMM in pellet (100x(HMM_pellet/(HMM_pellet+HMM_sup) | 12.11% | 4.95% | 6% |
| HMM lost to the centrifuge tube wall (calculated)^a^ and the associated unstirred layer | ~4% | ~3% | N/A |

^a^ The amount adsorbed to the wall was calculated from the internal surface area of the centrifuge tube and the HMM density of ~5000 HMM molecules µm^-2^ on both hydrophobic and hydrophilic surfaces after > 1 min incubation with HMM at 120 µg/ml (e.g. Persson et al., 2010; Sundberg et al., 2006).

Legends for supporting movies

**Movie S1. Representative in vitro motility assay on TMCS surface before affinity purification and without any blocking actin (from Fig. 2a, c).** The movie is shown at a frame rate of 4.98 frames per second. Temperature, 28.2°C. The field of view is 133.3 x 133.3 µm^2^.

**Movie S2. Representative in in vitro motility assay on TMCS surface after affinity purification without blocking actin (from Fig. 2a,c).** Frame rate, 4.98 frames per second. Temperature, 28.1°C. The field of view is 133.3 x 133.3 µm^2^.

**Movie S3. Representative in vitro motility assay on TMCS surface without affinity purification but with 1µM blocking actin (from Fig. 3a,c; Fig. S3).** Frame rate, 4.98 frames per second. Temperature, 28.4°C. The field of view is 133.3 x 133.3 µm^2^.

**Movie S4. Representative in vitro motility assay on TMCS surface after affinity purification and with 1µM blocking actin (from Fig. 4a,b; Fig. S2).** Frame rate, 4.98 frames per second. Temperature, 28.3°C. The field of view is 133.3 x 133.3 µm^2^.

**Movie S5. Representative in vitro motility assay on TMCS surface without affinity purification, without blocking actin and in the presence of 1 mM MgATP during HMM incubation (from Fig. 5a).** Frame rate, 4.98 frames per second. Temperature, 28.1°C. The field of view is 133.3 x 133.3 µm^2^.

**Movie S6. Representative in vitro motility assay on TMCS surface without affinity purification and without blocking actin but in the presence of 1 mM MgATP during HMM incubation (from Fig. 5a).** Frame rate, 4.98 frames per second. Temperature, 28.0°C. The field of view is 133.3 x 133.3 µm^2^.

**Movie S7. Representative in vitro motility assay on TMCS surface without affinity purification, without blocking actin and without 1 mM MgATP during HMM incubation (from Fig. 7).** Frame rate, 4.98 frames per second. Temperature, 28.4°C. The field of view is 133.3 x 133.3 µm^2^.

**Movie S8. Representative in vitro motility assay on TMCS surface without affinity purification and without blocking actin but in the presence of 1 mM MgATP during HMM incubation (from Fig. 7).** Frame rate, 4.98 frames per second. Temperature, 28.0°C. The field of view is 133.3 x 133.3 µm^2^.

**Movie S9. In vitro motility assay with Alexa-488 labelled actin filaments (added at 1 µM) propelled by HMM on TMCS derivatized surface as in a standard in vitro motility assay where the Alexa-labelled filaments take the place of blocking actin.** A snapshot of this Movie shown in Fig. S4. Frame rate, 4.98 frames per second. Temperature, 28.0°C. The field of view is 84 x 84 µm^2^.
